# Investigation into Propolis Components Responsible for Inducing Skin Allergy: Air Oxidation of Caffeic Acid and Its Esters Contribute to Hapten Formation

**DOI:** 10.1021/acs.chemrestox.2c00386

**Published:** 2023-05-15

**Authors:** Lorena Ndreu, Alexander K. Hurben, Gunnar S.A. Nyman, Natalia Y. Tretyakova, Isabella Karlsson, Lina Hagvall

**Affiliations:** † Department of Environmental Science, Stockholm University, Stockholm 114 19, Sweden; ‡ Department of Medicinal Chemistry and the Masonic Cancer Center, University of Minnesota, Minneapolis, Minnesota 55455, United States; § Department of Dermatology and Venereology, Sahlgrenska University Hospital, Region Västra Götaland, Gothenburg 413 45, Sweden; ∥ Department of Dermatology and Venereology, Institute of Clinical Sciences, Sahlgrenska Academy, University of Gothenburg, Gothenburg 405 30, Sweden; ⊥ Department of Occupational and Environmental Medicine, Lund University, Lund 22363, Sweden

## Abstract

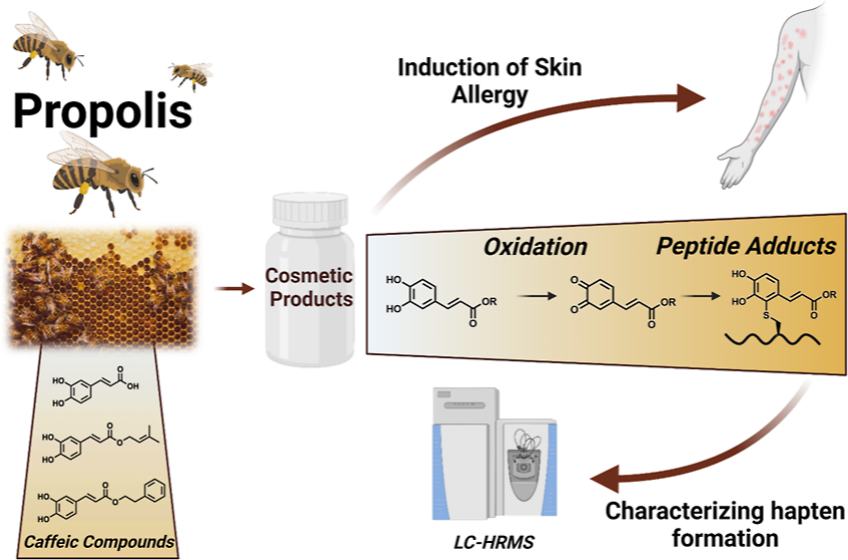

Propolis is a resin-like material produced by bees from the buds of poplar and cone-bearing trees and is used in beehive construction. Propolis is a common additive in various biocosmetics and health-related products, despite the fact that it is a well-known cause of contact allergy. Caffeic acid and its esters have been the primary suspects behind the sensitization potency of propolis-induced contact allergy. However, the chemical structures of the protein adducts formed between these haptens and skin proteins during the process of skin sensitization remain unknown. In this study, the reactivity of three main contact allergens found in propolis, namely, caffeic acid (CA), caffeic acid 1,1-dimethylallyl ester (CAAE), and caffeic acid phenethyl ester (CAPE), was investigated. These compounds were initially subjected to the kinetic direct peptide reactivity assay to categorize the sensitization potency of CA, CAAE, and CAPE, but the data obtained was deemed too unreliable to confidently classify their skin sensitization potential based on this assay alone. To further investigate the chemistry involved in generating possible skin allergy-inducing protein adducts, model peptide reactions with CA, CAAE, and CAPE were conducted and analyzed via liquid chromatography–high-resolution mass spectrometry. Reactions between CA, CAAE, and CAPE and a cysteine-containing peptide in the presence of oxygen, both in closed and open systems, were monitored at specific time points. These studies revealed the formation of two different adducts, one corresponding to thiol addition to the α,β-unsaturated carbonyl region of the caffeic structure and the second corresponding to thiol addition to the catechol, after air oxidation to o-quinone. Observation of these peptide adducts classifies these compounds as prehaptens. Interestingly, no adduct formation was observed when the same reactions were performed under oxygen-free conditions, highlighting the importance of air oxidation processes in CA, CAAE, and CAPE adduct formation. Additionally, through NMR analysis, we found that thiol addition occurs at the C-2 position in the aromatic ring of the CA derivatives. Our results emphasize the importance of air oxidation in the sensitization potency of propolis and shed light on the chemical structures of the resultant haptens which could trigger allergic reactions in vivo.

## 1 Introduction

Propolis is a resinous mixture produced by honeybees and used in beehive construction. Within the hive, propolis is deposited on the internal walls to serve as a thermal insulator and is also applied to holes and cracks to protect the hive from external invaders. Bees generate propolis by collecting plant exudates from poplar species and metabolize them by β-glycosidase, an enzyme present in bee saliva and beeswax. The final heterogeneous product is soft and sticky in warm temperatures and hard when cool, which is critical for its structural functions.^1^ Alongside its mechanical attributes, bees utilize the antibacterial, antimycotic, and antiviral properties of propolis for nest protection.^2^ These medicinal features of propolis have prompted its widespread use by humans for thousands of years. Today, propolis is used in many consumer products including dietary supplements, health-related products, and biocosmetics, resulting in widespread human exposure.^1^

The chemical composition of propolis can vary dramatically, both qualitatively and quantitatively,^3^ depending on the plant used for its production,^2^ the season it was collected,^4^ the race of honeybees,^5^ and the method used to harvest propolis.^6^ However, a majority of crude propolis contains 50–70% resin, 30–50% oils and waxes, 5–10% pollen, and other minor chemical compounds such as amino acids, sugars, vitamins B, C, and E, minerals, flavonoids, phenols, and aromatic compounds such as caffeic acid (CA), caffeic acid 1,1-dimethylallyl ester (CAAE), and caffeic acid phenethyl ester (CAPE).^7^

Given its widespread use, it is concerning that propolis is a well-known cause of contact allergy. Accordingly, propolis has been recently added to the test battery of compounds used in routine diagnosis of allergic contact dermatitis (ACD) in Europe.^8,9^ Skin sensitization and its clinical manifestation, ACD, are caused by small reactive compounds (haptens) which form immunogenic complexes in reaction with proteins in the skin. Prehaptens are compounds which need activation, such as oxidation, in order to become reactive toward proteins. Mechanistically, contact allergy is an acquired immunological memory of the protein adducts induced by haptens. Characterizing the molecular initiation events, i.e., the modification or haptenation of skin proteins by reactive compounds, is of utmost importance in understanding the process of skin sensitization.

With regard to propolis, it is believed that CA and its esters are the primary chemical species responsible for its haptenic activity and allergenicity.^10,11^ This has led to various investigations assessing the role of these compounds in ACD. In a recent study of patients suffering from cheilitis (eczema of the lips), we investigated the importance of CA, CAAE, and CAPE in contact allergy to propolis and beeswax.^12^ Out of 10 patients with contact allergy to beeswax, none were found to react to CA, while 3 of the patients reacted to both CAAE and CAPE, suggesting that these compounds may play a role in ACD to beeswax and propolis. In a similar study, Hausen investigated contact allergy to four CA esters, including CAPE, in 27 patients previously sensitized to propolis.^11^ CAPE produced immune reactions in 75% of patients, providing additional evidence that CA esters are important contributors in ACD to propolis.

The molecular mechanisms responsible for the allergenic activity of CA and CA esters are not fully understood. CA derivatives harbor two reactive sites, an α,β-unsaturated carbonyl, which serves as a Michael acceptor, and a catechol, which is prone to oxidation to a reactive o-quinone. Therefore, it has been hypothesized that these compounds act as haptens or possibly prohaptens.^13^ However, the structures of CA and CAPE-induced protein and peptide adducts have not been elucidated. Only the adduct between CAAE and reduced glutathione has been reported.^13^ Assessing the reactivity of CA and CA esters in controlled model systems is a critical step toward deciphering how these compounds induce skin sensitization and elicit ACD.

**Figure 1:**
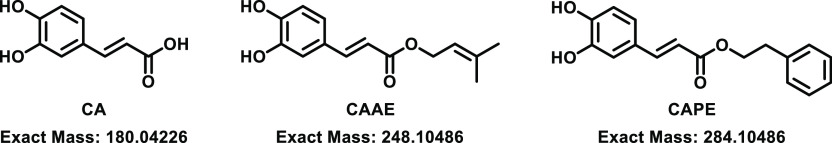
Structures and exact mass of CA, CAAE, and CAPE.

Here, we investigated the protein reactivity of three suspected sensitizers present in propolis, namely, CA, CAAE, and CAPE (Figure 1). Initially, a kinetic direct peptide reactivity assay (kDPRA) was attempted to classify the caffeic acid derivatives’ sensitization potency. Next, the possibility of catechol air oxidation to o-quinone was evaluated through a series of experiments and reactions with a cysteine-containing peptide Ac-PHCKRM. The nature of the peptide adducts formed from each compound was examined by liquid chromatography–high-resolution mass spectrometry (LC-HRMS) and nuclear magnetic resonance (NMR) spectroscopy. It was observed that the formation of adducts through the aromatic ring systems in CA, CAAE, and CAPE occurred two to five times faster than the adducts generated at the α,β-unsaturated carbonyl moieties and that the presence of oxygen was necessary for peptide adduct formation. This information provides insight into CA, CAAE, and CAPE adduct formation chemistry and identifies possible haptens responsible for triggering ACD in individuals with contact allergy to propolis.

## 2 Materials and Methods

### 2.1 Chemicals and Materials

CA, CAAE, CAPE, cinnamic aldehyde, sodium phosphate monobasic monohydrate (NaH_2_PO_4_ H_2_O), sodium phosphate dibasic heptahydrate (Na_2_HPO_4_ 7H_2_O), 1,1-diphenyl-2-picrylhydrazyl radical (DPPH), NaIO_4_, KIO_4_, 2,4-dinitrochlorobenzene (DNCB), and *N*-acetylcysteine (NAC) were purchased from Sigma-Aldrich (Steinheim, Germany). Deuterated NMR solvents were acquired from Cambridge Isotope Laboratories. Ethanol (EtOH) (96%) and formic acid (FA) (99–100%) were obtained from VWR International (Pennsylvania, US). Acetonitrile (ACN) (≥99.9%), LC-grade water, and the 2 mL deep-well plates were acquired from Thermo Fisher Scientific (Seelze, Germany). N-Terminal acetylated peptide Ac-PHCKRM was obtained from Peptide 2.0 (Chantilly, US), while Ac-RFAACAA was acquired from RS Synthesis (Louisville, United States). Monobromobimane (MBrB) was purchased from Cayman Chemical (Michigan, United States). 96-well black plates were purchased from PerkinElmer (Massachusetts, United States).

### 2.2 Modified Kinetic Direct Peptide Reactivity Assay

#### 2.2.1 Assay Protocol

Stock solutions of CA, CAAE, and CAPE were prepared fresh. Initial solutions were prepared in ACN at a concentration of 20 mM. These were used to prepare a dilution series of 20, 10, 5, 2.5, and 1.25 mM for each compound. 120 μL of a 0.667 mM peptide (Ac-PHCKRM) stock solution in pH 7.4 phosphate buffer (PB)/ACN were added to a 96-well plate. Next, 40 μL of compounds were added to the wells to provide a final 0.5 mM peptide concentration and 5, 2.5, 1.25, 0.625, and 0.3125 mM, respectively, of the tested compounds. The final volume in each well was 160 μL, and the final composition of the incubations was PB (pH 7.4)/ACN (50:50) to accommodate potential solubility issues. The plates were sealed with a gas-tight adhesive foil and shaken for 5 min, and the reactions were allowed to proceed for 10, 30, 90, 150, 210, or 300 min at 25 ± 2.5 °C. The reactions were stopped by addition of 40 μL of a 3 mM solution of MBrB, which reacts rapidly with unmodified cysteine moieties of the peptide Ac-PHCKRM to generate a fluorescent complex. The plate was shaken for 5 min prior to measuring fluorescence with a SpectraMax iD3 plate reader from Molecular Devices (Ex: 390 nm, Em 480 nm). DNCB, a known extreme skin sensitizer causing peptide depletion with a reproducible rate constant was used as a positive control. Other controls included peptide only as a negative control (NC) and respective test substance only as a substance control (SC).

#### 2.2.2 Data Evaluation

The relative peptide depletion (DP) % was calculated for each compound tested through eq 1.
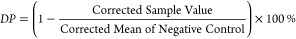
1

The corrected sample value is taken as the measured sample fluorescence value minus the value of the test SC. The corrected mean value of the NC is the average of 12 NC measurements minus the mean of 12 background measurement controls. The obtained DP % values are then converted to 1n(100-DP) for kinetic analysis. The slope and correlation values are then generated from plots of 1n(100-DP) versus the different concentrations for 10, 30, 90, 150, 210, and 300 min reaction time points.

### 2.3 LC-HRMS Analysis of CA, CAAE, and CAPE Peptide Reactions

#### 2.3.1 Influence of Oxygen on Peptide Depletion and Adduct Formation

##### 2.3.1.1 Reaction in a Closed System in the Presence of Oxygen

Fresh stock solutions of CA, CAAE, CAPE, and the Ac-PHCKRM peptide were prepared, and each compound in 5-fold molar excess (250 nmol) was incubated with the peptide (50 nmol) in a 1:1 mixture of PB (pH 7.4) and ACN directly in LC vials. The final volume of each incubation was 1 mL; the final concentration of each tested compound was 250 μM and that of the peptide was 50 μM. LC-HRMS analysis was conducted at six different time points, which were 1 min, 1 h, 2 h, 3 h, 4 h, and 5 h. Incubation of the peptide alone was used as a NC.

##### 2.3.1.2 Reaction in a Closed System under an Inert Atmosphere (Oxygen-Free)

Fresh stock solutions of CA, CAAE, CAPE, and the Ac-PHCKRM peptide were prepared and purged with argon for 30 min. Incubations of each compound in 5-fold molar excess (250 nmol) with the peptide (50 nmol) were prepared directly on LC vials in a mixture of PB (pH 7.4) and ACN at a 1:1 ratio. The final volume of each incubation was 1 mL; the final concentration of each tested compound was 250 μM and that of the peptide was 50 μM. The incubations were analyzed by LC-HRMS at 1 min, 1, 2, 3, 4, and 5 h time points. All incubations were purged for 1 min after preparation. Incubation of the peptide alone was used as a NC.

##### 2.3.1.3 Reaction in an Open System (Ambient Air)

Stock solutions of CA, CAAE, CAPE, and the Ac-PHCKRM peptide were made fresh. Incubations of the peptide (250 nmol) with each tested compound at 5-fold molar excess were prepared in a 5 mL final reaction volume consisting of PB (pH 7.4) and EtOH in a 1:1 ratio. The final concentration of each tested compound was 250 μM and that of the peptide was 50 μM. The reaction mixtures were incubated for 5 h in open air upon constant stirring. Sample aliquots (100 μL) were collected from each incubation after 1 min, 1, 2, 3, 4, and 5 h and analyzed directly by LC-HRMS to obtain structural information about the adducts formed with the trapping peptide. Incubation of the peptide without the test substance was used as a NC.

##### 2.3.1.4 LC-HRMS Instrumental Parameters

All LC-ESI-HRMS analyses were performed on a Dionex UltiMate 3000 UHPLC coupled to a Q-Exactive Quadrupole-Orbitrap mass spectrometer equipped with an electrospray ionization (ESI) source. Chromatographic separation was achieved on an AcclaimTM RSLC 120 C18 (2.1 × 150 mm i.d., particle size 2.2 μM, Thermo Scientific, Sunnyvale, CA) column. The mobile phases were Milli-Q-water containing 0.1% FA (A) and ACN containing 0.1% FA (B). The gradient program used started at 5% B (0–2 min), 5–60% B (2–10 min), 60–95% B (10–10.01), 95% B (10.01–12.5 min), 95–5% B (12.5–12.51), and 5% B (12.51–15 min). The flow rate was set to 0.3 mL/min, and the injection volume was 2 μL. The instrument was operated in the positive ion mode using the parallel reaction monitoring (PRM) scan mode. The expected chromatographic peak width was 15 s. PRM experiments were performed at a resolution of 30,000, an AGC target of 2 × 10^5^, a maximum IT of 100 ms, an isolation window of 0.5 *m*/*z,* and a normalized collision energy of 30. The inclusion list of the PRM method was based on the masses expected from the reaction of each compound with the peptide Ac-PHCKRM, both singly and doubly charged, as well as the mass-to-charge ratios of the singly and doubly charged unmodified peptide, Table S1.

### 2.4 NMR Analysis of CA, CAAE, and CAPE NAC Reactions

Fresh stock solutions of CA, CAAE, CAPE, NaIO_4_, and NAC were prepared in a 1:1 mixture of deuterated phosphate-buffered saline (PBS) (pH 7.4, pD uncorrected) and MeOD (d4) or DMSO (d6). Deuterated PBS was prepared through lyophilizing PBS and reconstituting the resultant powder with D_2_O; this process was repeated three times. Reactions were initiated by combining the specified ratios of reactants. Spectra were obtained on a 500 MHz Bruker spectrometer. Reactions were monitored with a ^1^H pulse sequence using a data accumulation of 16 scans for each spectrum.

### 2.5 UV–Vis Monitoring of CA, CAAE, and CAPE Air Oxidation

Fresh stock solutions of CA, CAAE, CAPE, and KIO_4_ were prepared in a 1:1 mixture of PBS (pH 7.4 or pH 8.8) and EtOH. Reactions were initiated by combining equimolar ratios of reactants at a final concertation of 50 μM in a 96-well plate at a final volume of 100 μL. The spectra were acquired on a SpectraMax iD3 plate reader from Molecular Devices.

### 2.6 DPPH Reactions with CA, CAAE, and CAPE

Solutions containing 0.8 mM CA, CAAE, and CAPE were prepared fresh in EtOH/H_2_O (1:1). An equimolar DPPH solution was prepared fresh in EtOH. Equal volumes of DPPH and tested compounds were combined in a 96-well plate, and the absorbance at 524 nm was measured every 10 min over a period of 1 h. Absorbance measurements were obtained on a SpectraMax iD3 plate reader from Molecular Devices.

## 3 Results

### 3.1 Classification of CA, CAAE, and CAPE Skin Sensitization Potency

Our first objective was to test the skin sensitization potential of CA, CAAE, and CAPE. The direct peptide reactivity assay (DPRA) has become the standard method to assess the small-molecule sensitization potential in accordance with the OECD test guideline 442C established in 2015.^14^ At present, this technique is commonly employed for assessing skin-sensitizing potential since animal-based in vivo testing was prohibited in cosmetic testing in Europe in 2013. The DPRA measures the depletion of a synthetic cysteine- and a lysine-containing peptide following 24 h of incubation with a single concentration of a test substance. Recently, a modified version of the DPRA assay has been introduced, the kDPRA, where various concentration and timepoint measurements have been incorporated in the assay design to provide kinetic information about the reactivity of a particular test compound. The kDPRA utilizes a cysteine reactive dye, mBrB, to measure the concentration of a cysteine-containing peptide used in the assay. mBrB generates a fluorescent complex upon reaction with any remaining unmodified cysteines not depleted by the test compounds. The fluorescence signal can then be used to determine the remaining concentration of nondepleted peptide (DP) and DP %.

**Figure 2:**
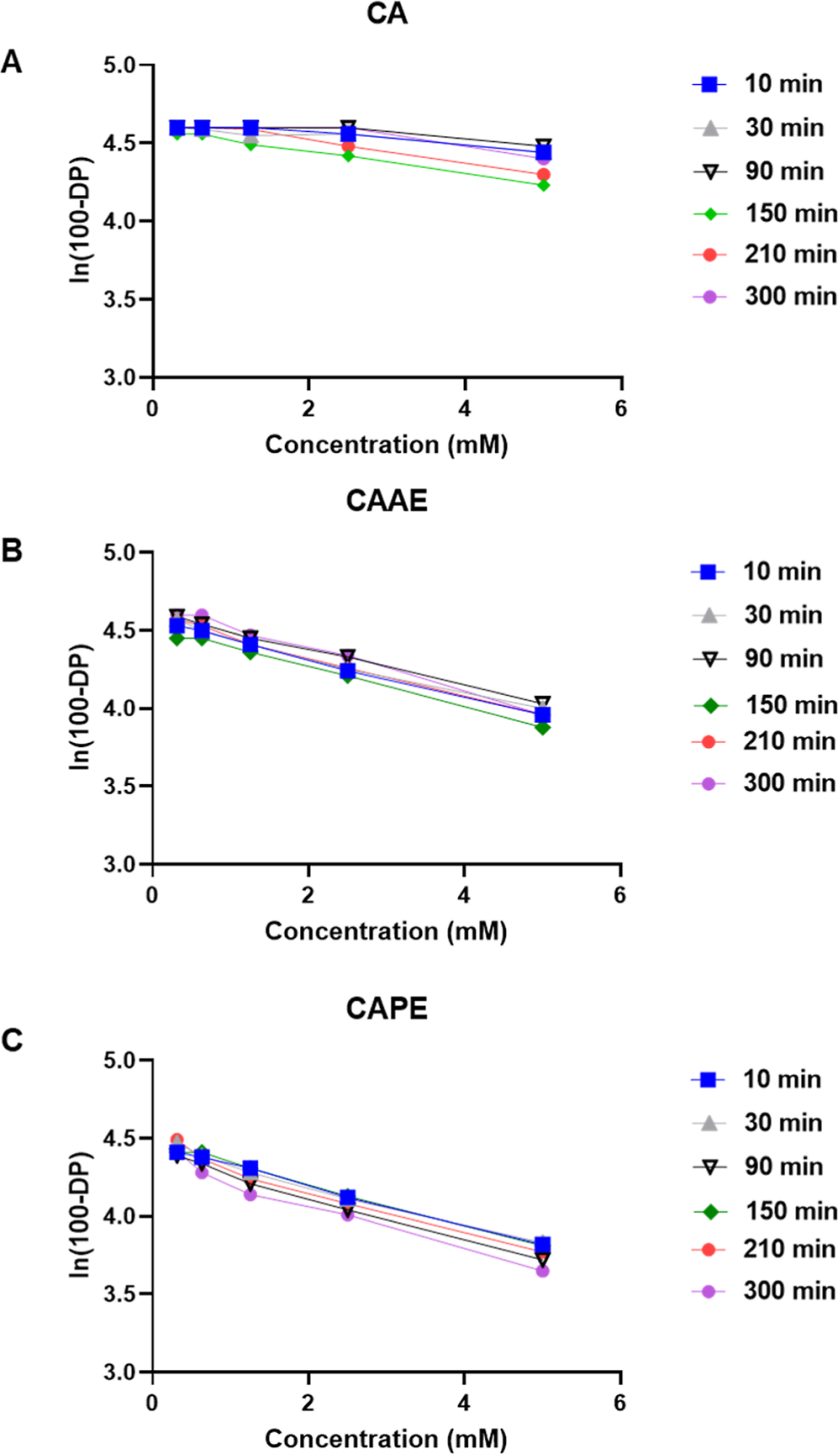
Modified kDPRA results for (A) CA, (B) CAAE, and (C) CAPE. The natural log of unreacted Ac-PHCKRM peptide [100—relative peptide depletion (DP) %] values are plotted against the increasing concentrations of CA, CAAE, and CAPE at specified time points.

Initially, the kDPRA was performed according to the OECD guidelines (see the complete protocol described in the Supporting Information). However, as seen in Figure S1, no significant changes for ln(100-DP) were observed with respect to CA, CAAE, and CAPE incubation time, which disallowed accurate conclusions regarding sensitization potential based on first-order kinetic rate assumptions to be drawn with these data. A modified version of the assay was then performed in a second attempt to classify the sensitization potential of these compounds. Changes included using a different cysteine-containing peptide (Ac-PHCKRM), a final solvent composition of 1:1 PB (pH 7.4)/ACN vs 3:1 of the original protocol in an effort to improve the solubility of the CA compounds, and a shorter final incubation time point (300 min vs 1440 min of the original protocol). However, even this second approach exhibited no significant variation in slope for ln(100-DP) with respect to incubation time (Figure 2). Thus, the lack of expected first-order kinetics prohibited using assumptions in the kDPRA to classify the skin sensitization potential of the three tested compounds. However, the data did indicate that CAPE and CAAE reacted faster than CA. The challenges encountered during the two kDPRA approaches dictated the need to employ a more sensitive technique to compare the peptide depletion caused by the tested compounds and investigate the structure of the adducts formed in each case.

### 3.2 Structural Analysis of CA, CAAE, and CAPE Peptide Adducts

**Figure 3:**
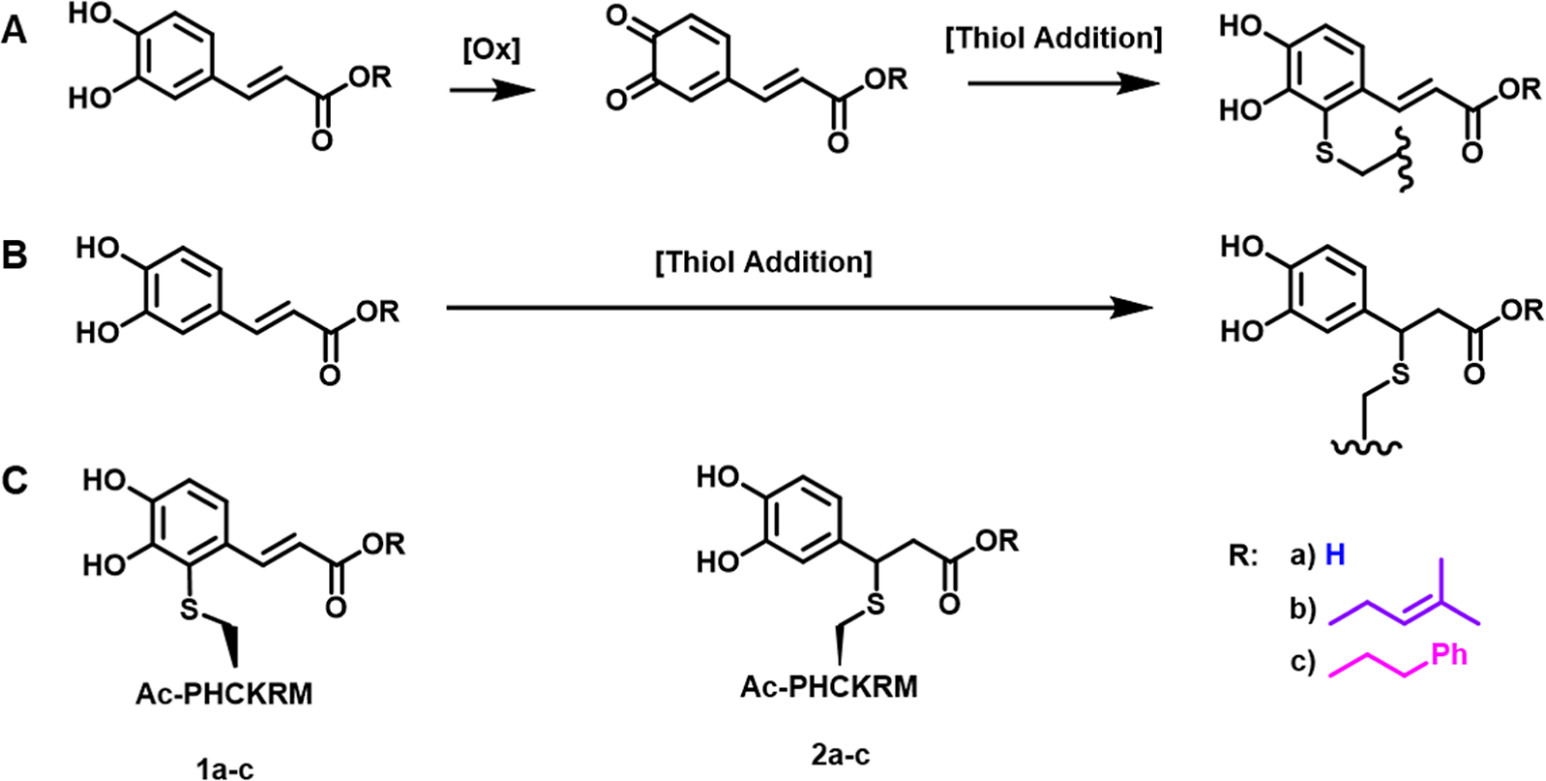
CA, CAAE, and CAPE peptide adduct formation. (A) Catechol oxidation to o-quinone followed by thiol addition. (B) Thiol addition to the α,β-unsaturated carbonyl (C) Structures of CA, CAAE-, and CAPE-derived peptide adducts.

The synthetic peptide Ac-PHCKRM was subjected to three different incubation conditions with a 5-fold molar excess of the tested compounds, and the resulting mixtures were analyzed by LC-HRMS. These incubations were conducted in closed systems under both aerobic (where free and/or dissolved oxygen was present) and anaerobic conditions (argon atmosphere), as well as in an open system (ambient air), with constant stirring. The aim was to assess the influence of oxygen in adduct formation and to elucidate the structure of the peptide adducts generated by these compounds to gain insight into the nature of potential haptens formed in vivo. The peptide used harbors cysteine, lysine, and histidine side chains which have been reported to react with o-quinones.^15^ Additionally, cysteine residues can undergo Michael addition reactions with α,β-unsaturated carbonyls, which leads to an array of potential adducts (Figure 3).

**Figure 4:**
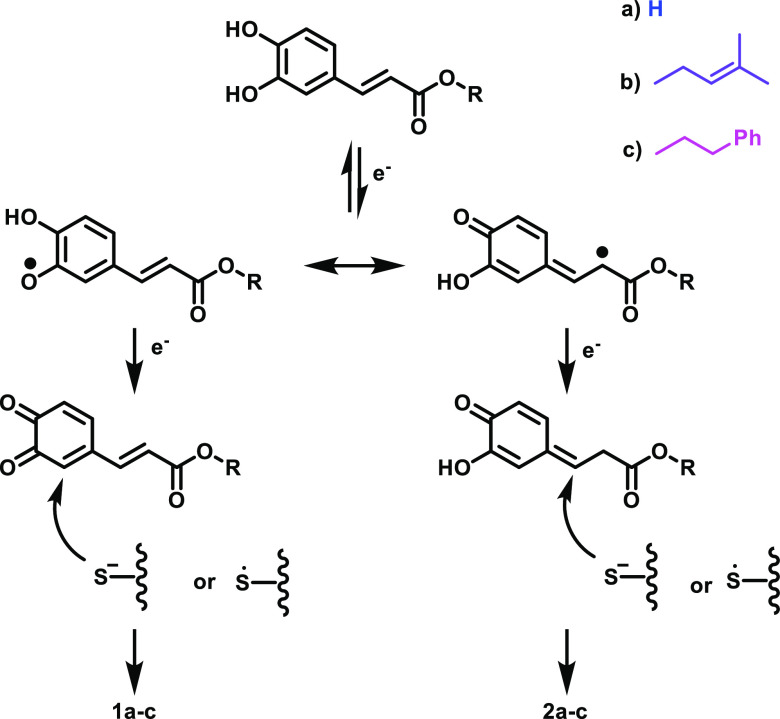
Tentative mechanisms for generation of caffeic acid derivative adducts through thiolate and thiyl radical pathways.

Furthermore, cysteines are also prone to thiyl radical addition.^16−18^ The predicted masses of the expected adducts are shown in Table S3. LC-ESI-MS/MS data makes it possible to elucidate the type of reactive species involved in adduct formation (from molecular mass) and to determine the peptide site of modification from characteristic b- and y-ions produced during MS2 fragmentation. Specifically, CA and its derivatives could react with cysteine residues of the proteins directly via Michael addition (Figure 3B) or following oxidation of catechol to a quinone species (Figure 3A). It is also possible that the generation of radicals during oxidation can lead to the formation of adducts **2a–c** via a thiol–ene mechanism.^19^ An overview of potential reaction mechanisms of thiolate and thiyl radicals additions is shown in Figure 4 which highlights the multiple pathways CA, CAAE, and CAPE could undergo to generate adducts.

**Figure 5:**
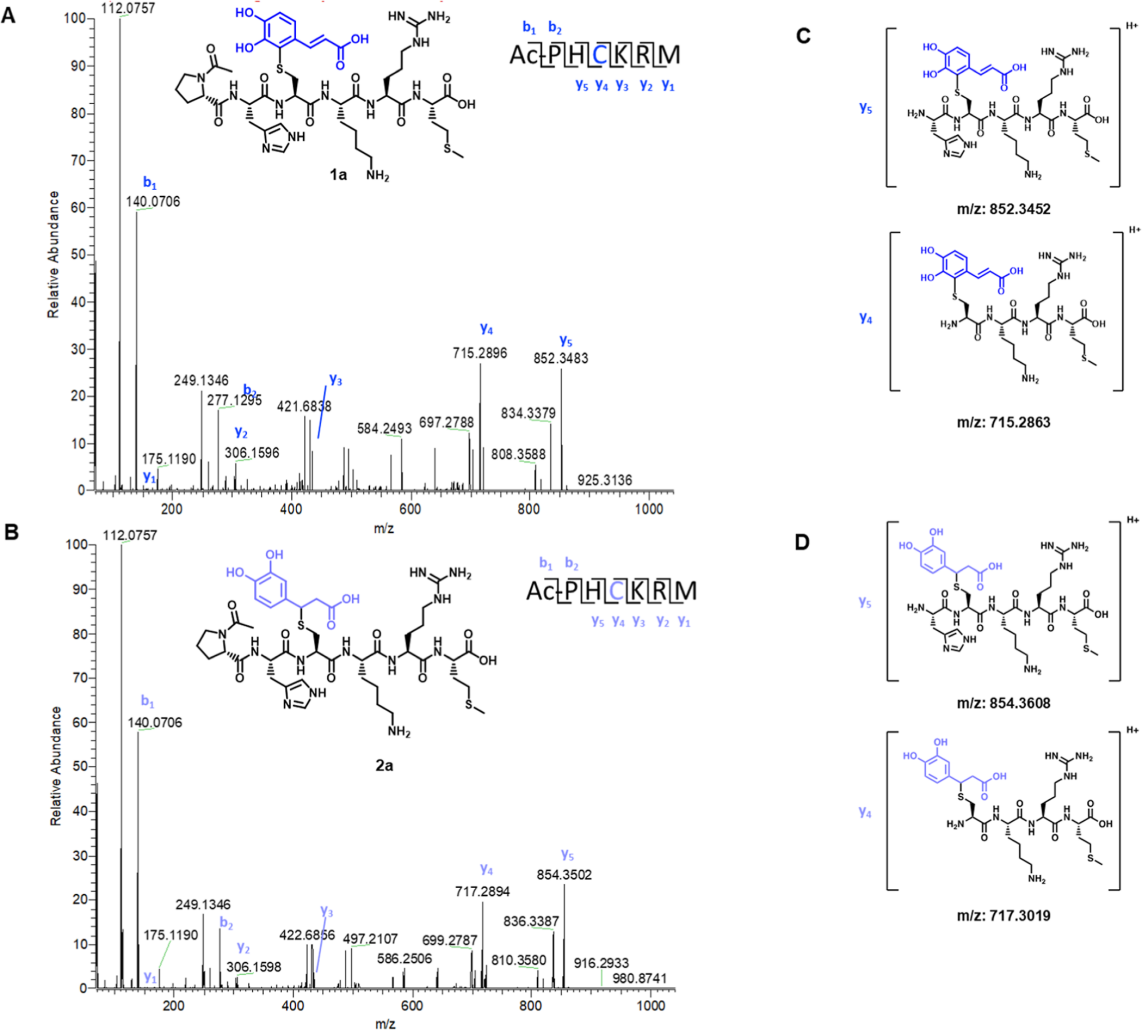
LC–ESI^+^–HRMS structural analysis of CA and Ac-PHCKRM peptide adducts after incubation of the peptide with 5 times molar excess of CA, in 1:1 PB (pH 7.4) ACN for 5 h prior to LC-HRMS analysis. (A,B) MS^2^ spectrum of **1a** and **1b** with annotated b and y ions. (C,D). Structures of the y ions carrying the CA adduct.

CA, CAAE, and CAPE peptide adduct formation in all incubations was analyzed at hour increments by LC-HRMS. Representative MS^2^ spectra for CA-generated adducts after a 5 h incubation are shown in Figure 5. Analogous chromatograms and spectra for CAAE and CAPE peptide adducts can be found in the Supporting Information (Figures S2 and S3). LC–MS/MS data revealed that incubation of CA, CAAE, and CAPE with Ac-PHCKRM, in the presence of oxygen both in a closed and open system, led to the formation of two different adducts, one corresponding to addition into the aromatic ring (**1a**, **1b**, and **1c**, respectively) and the other corresponding to the addition into the α,β-unsaturated carbonyl (**2a**, **2b**, and **2c**, respectively). We also verified via NMR that thiol addition to the ring system occurs at the 2-position through the reaction of oxidized CA and NAC (Figure S4). This finding is in agreement with previous reports.^13^ By MS, these two sets of adducts are distinguishable by the 2 *m*/*z* difference between the singly charged ion species and the 1 *m*/*z* difference between the doubly charged species. The MS^2^ b- and y-ions series verified that the reaction of CA, CAAE, and CAPE with the Ac-PHCKRM peptide occurs with the cysteine residue under these reaction conditions, suggesting that cysteine could be the main amino acid involved in hapten–protein reactions of CA derivatives in vivo.

### 3.3 Influence of Oxygen in Peptide Depletion and Adduct Formation

For all incubations, we utilized LC-HRMS to compare the rates of adduct formation at the aromatic ring versus at the α,β-unsaturated carbonyl. This was accomplished by integrating the peak areas of the extracted ion chromatogram corresponding to each respective adduct as well as an unreacted peptide to calculate relative ratios of adduct vs unreacted peptide at each time point. The impact of oxygen on adduct formation was examined by conducting similar reactions in three distinct environments: a closed system with limited oxygen excess, an open-air system with excess oxygen availability under stirring, and a closed system with an inert atmosphere without oxygen. A reaction buffer composed of a 1:1 mixture of PB (pH 7.4)/ACN was selected to keep the reaction conditions similar to the kDPRA conditions. In the ambient air reactions, however, a buffer comprising PB (pH 7.4)/EtOH (1:1) was chosen since CA has a higher solubility in EtOH than ACN.

**Figure 6:**
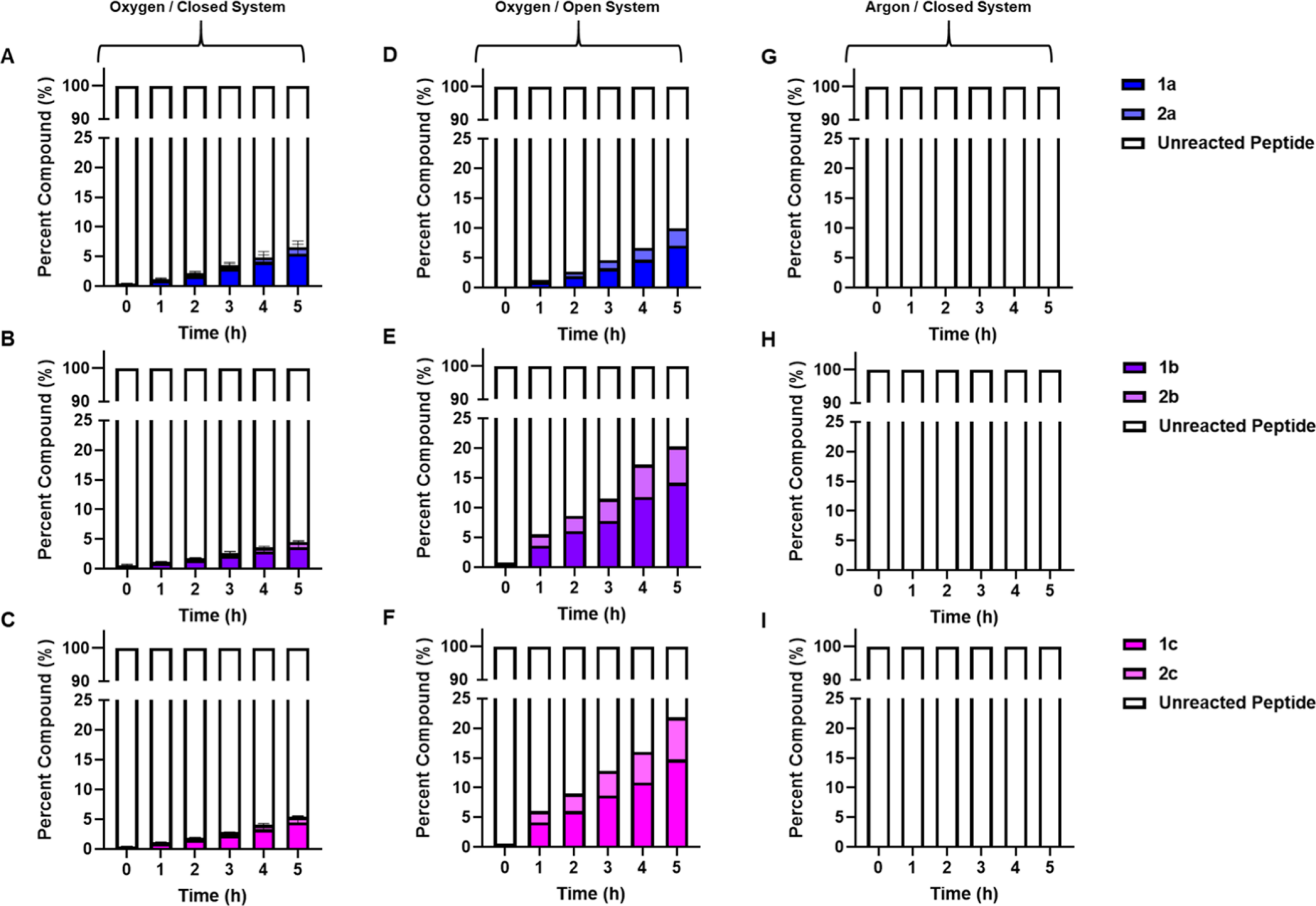
Relative ratios of peptide adducts with CA, CAAE, and CAPE. The model peptide was incubated with five times molar excess of each compound in the presence of oxygen, in a closed system and open system (ambient air), and under an argon atmosphere prior to LC-HRMS analysis. Peak areas corresponding to each respective adduct and unreacted peptide were summed and used to calculate the relative percent compound present at each time point. (A,D,G) CA reactions. (B,E,H) CAAE reactions. (C,F,I) CAPE reactions.

As shown in Figure 6, adduct formation levels increased over time when the model peptide was incubated with all tested compounds in the presence of limited amounts of oxygen within a closed system. The levels of adduct formation between the CA, CAAE, and CAPE were not significantly different in the closed system. Cysteine addition to the ring system of the CA derivatives was found to be the most prominent adduct species in comparison to adducts formed at the α,β-unsaturated carbonyl by approximately fivefold. In an open-air system under constant stirring, adduct formation levels also increased over time. Cysteine addition to the ring system of the CA derivatives was once again observed to be the most prominent adduct species but only approximately 2-fold higher compared to adducts formed at the α,β-unsaturated carbonyl. The higher excess of adducts formed via cysteine addition to the ring system of the CA derivatives in the presence of oxygen indicates that thiol addition into the aromatic ring is kinetically favored over addition to the α,β-unsaturated carbonyl. In the closed system in the presence of oxygen, no significant difference could be observed for the two different adducts formed for all the compounds tested. However, such a difference in adduct formation levels could be observed in the CA incubations compared to CAAE and CAPE in the open-air system. As shown in Figure 6D–F, the adducts **1a**, **1b**, and **1c** compose 7, 14, and 15% of the reaction mixture after 5 h, whereas **2a**, **2b**, and **2c** make up 3, 6, and 7% of the final mixture, respectively. This ratio is mirrored in earlier reaction time points as well, indicating that air oxidation of CA derivatives to o-quinones is an important mechanism in the generation of this class of haptens. NMR analysis of the reaction mixtures between the caffeic acid derivatives and NAC also revealed that CAAE was more reactive than CA through the observation of resultant adducts (Figure S5).

To further examine the role air oxidation of CA, CAAE, and CAPE plays in peptide adduct formation, we performed analogous reactions to those described above but in an oxygen-free environment to prevent o-quinone formation. No peptide adducts were observed under these conditions as shown in Figure 6G–I. These results suggest that air oxidation processes likely play a critical role in the sensitization potential of these compounds.

### 3.4 Air Oxidation of CA, CAAE, and CAPE

**Figure 7:**
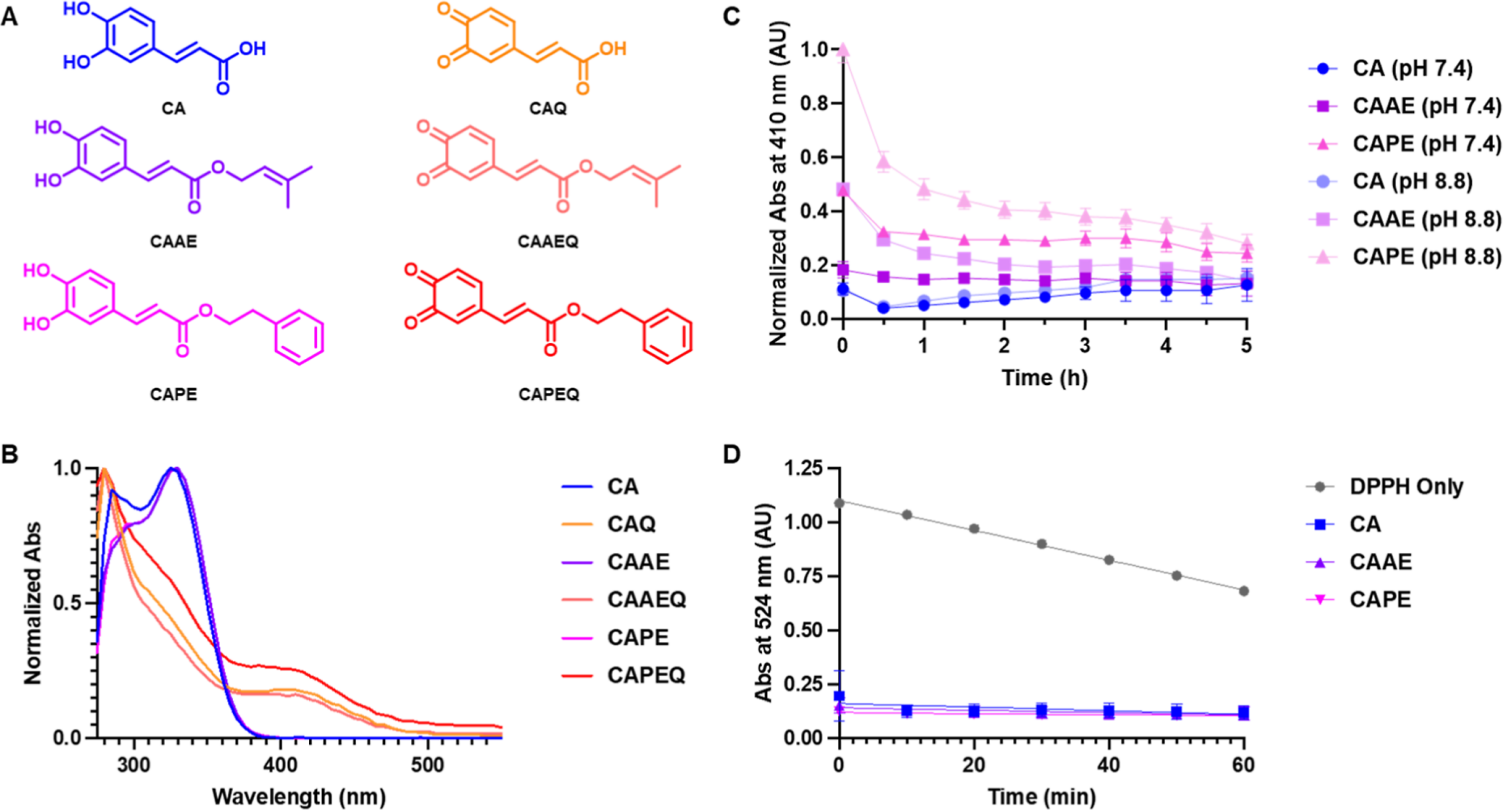
Oxidation propensity of CA, CAAE, and CAPE. (A) Structures of CA, CAAE, and CAPE paired with their respective o-quinones CAQ, CAAEQ, and CAPEQ. (B) Normalized UV absorbance spectra of CA, CAAE, and CAPE (50 μM) alone or after a 1 h incubation with equimolar KIO_4_ in a solution of 1:1 PB (pH 7.4): EtOH. (C) Absorbance at 410 nm taken at various time points of CA, CAAE, and CAPE (50 μM) solutions in 1:1 PB (pH = 7.4 or 8.8): EtOH. Data are normalized to CAPE abs values and are represented as mean ± SD from three replicates. (D) Absorbance at 525 nm taken at various time points of DPPH (0.8 mM) alone or in the presence of equimolar CA, CAAE, and CAPE in a 1:1 EtOH: H_2_O solution. Data are represented as mean ± SD from three replicates.

Given the apparent importance of CA, CAAE, and CAPE oxidation prior to peptide adduct formation, we sought to verify that these compounds were prone to air oxidation. The structures of the o-quinone of CA (CAQ), o-quinone of CAAE (CAAEQ), and o-quinone of CAPE (CAPEQ) are shown in Figure 7A. We took advantage of the characteristic UV–vis signatures of o-quinones to track catechol oxidation in the presence of oxygen.

First, we oxidized CA, CAAE, and CAPE, with KIO_4_ to establish reference UV–vis spectra of CAQ, CAAEQ, and CAPEQ as shown in Figure 7B. As expected, CAQ, CAAEQ, and CAPEQ gave new local UV maxima at 410 nm, diagnostic of o-quinone formation. Next, CA, CAAE, and CAPE solutions in a 1:1 mixture of EtOH/PBS at pH 7.4 or pH 8.8 were prepared and the absorbance at 410 nm was monitored over 5 h to assess the rate of oxidation and stability of the resultant quinones at physiological and elevated pH. As shown in Figure 7C, addition of CAAE and CAPE into the buffered solution immediately produced a colorimetric change at 410 nm which decayed over time, indicating that the resultant o-quinones are unstable. Reactions conducted at pH 8.8 as opposed to pH 7.4 led to an approximately 2-fold increase in absorbance at 410 nm, suggesting that high pH increases the rate of quinone formation.^20^ The apparent half-life of these quinones as calculated by a one-phase decay function was ∼0.4 h for CAAE and CAPE at pH 8.8. Reactions with CA yielded negligible differences between pH 7.4 and 8.8 which indicates that CA is less susceptible to air oxidation as compared to CAAE and CAPE. This result is in accordance with the lower reactivity observed in the clinical studies discussed above.^12^

Additionally, we probed the possibility that radical pathways may be involved in CA, CAAE, and CAPE air oxidation. The reagent, DPPH, is a stable radical that can abstract labile hydrogens from a suitable donor molecule and form a hydrazine derivative which produces a detectable colorimetric change. Thus, DPPH can determine if hydrogens susceptible to abstraction are present in CA, CAAE, and CAPE and provide an indication of their propensity to form radical species and, subsequently, their oxidation potential.^21,22^ We reacted an equimolar amount of DPPH with CA, CAAE, and CAPE and followed the reactions spectroscopically for 1 h. As shown in Figure 7D, all three compounds reacted instantaneously with DPPH, marked by the decrease in 524 nm absorbance. No measurable distinction could be made between CA, CAAE, and CAPE in our experimental conditions, which indicates that they all have hydrogens susceptible to abstraction, leading to radical formation and oxidation. These results suggest that air oxidation and possibly radical pathways as shown in Figure 4 drive hapten formation of CA derivatives.

## 4 Discussion

Propolis is a common ingredient in cosmetic and natural products and a common cause of contact allergies. The skin-sensitizing potential of propolis was first demonstrated in 1977 with the guinea pig maximization test. In that study, Petersen et al. found propolis to be a potent sensitizer in 19 out of the 25 animals used.^23^ Later, propolis and the extract, LB-1, a major constituent of poplar plant buds and poplar-type propolis, were found to be strong sensitizers through the complete adjuvant test in guinea pigs.^24^ The composition of LB-1 was later characterized as a mixture of 54.2% CAAE, 28.3% caffeic acid 3-methyl-3-butenyl ester (CA3M3BE), 7.9% CAPE, 4.3% 2-methyl-2-butenyl ester of caffeic acid (CA2M2BE), 1.3% CA, and 1.0% benzyl ester of caffeic acid (CABE), suggesting that caffeic acid derivatives may contribute to ACD.^25^ Subsequent investigations of 26 compounds found in propolis and/or poplar buds, also classified CA esters as strong sensitizers.^10,24−26^ Such findings warrant further investigation into the chemistry of CA and esters of CA, such as CAAE and CAPE, to elucidate the structure and the mechanisms of formation of potential haptens responsible for triggering skin sensitization and ACD upon propolis exposure.

Attempts to classify the tested compounds using the kDPRA were not successful. Both the data obtained from the OECD procedure (Figure S1) and the modified version tested (Figure 2) showed no significant variation in the reaction rate with respect to time, inconsistent with the clinical data discussed earlier, especially for CAPE. From the data, only the concentration of the tested compounds was shown to play a role in the adduct formation rate. The limitations of the kDPRA, including the case of compounds where oxygen is crucial for the formation of short-lived highly reactive species, are discussed in a critical and thorough review by Roberts.^27^ For these types of compounds, four stages of the chemical reactions taking place during the assay are described. The first stage involves the presence of highly reactive oxidation products before the start of the assay. The second stage involves a short induction period where highly reactive species build up. The third stage involves the rate of peptide depletion becoming dependent on the concentration of the test material and oxygen in the reaction medium. The fourth and final stage involves the mass transfer of oxygen from the atmosphere to the reaction solution, which becomes the rate-determining step after most of the oxygen in the solution has become depleted. Thus, the use of the kDPRA to assess sensitization potential may not be applicable to all classes of potential haptens and should be carefully applied to avoid misclassification of compounds’ sensitization potential. Given these results, we sought to investigate the reactivity of CA, CAAE, and CAPE and the influence of oxygen in their adduct formation by means of LC-HRMS.

To date, few studies have attempted to determine the structures of CA-, CAAE-, and CAPE-derived peptide adducts.^13^ Here, our LC-HRMS data show that cysteine is the most reactive amino acid toward these caffeic acid derivatives as adducts were formed exclusively at this residue. This is in line with previous reports which measured the rate of thiol addition to o-quinones to be 10,000 times faster than that of amine addition.^28^

Although the classification of the three tested compounds with the kDPRA was not successful, utilization of LC-HRMS revealed time-dependent CA, CAAE, and CAPE peptide adduct formation in the presence of oxygen. However, only under open-air conditions, could discrimination on adduct levels formed from CA compared to CAAE and CAPE be made. In a closed system with a limited amount of oxygen, CA, CAAE, and CAPE generated similar amounts of adducts. However, CAAE and CAPE generated twice as many peptide adducts compared to CA in open-air conditions. We hypothesize that deprotonation of the free carboxylic acid of CA to the carboxylate anion increases the electron density within its conjugated system, thereby lowering its electrophilicity in comparison to its ester derivatives. In the context of ACD, the open-air system is more representative of the systems used during patch testing, as well as of the biological systems these compounds experience in vivo.

We also found that peptide adducts to the aromatic ring system of CA, CAAE, and CAPE were five times more abundant than those formed via addition to the α,β-unsaturated carbonyl when reactions were performed in a closed system. In an open-air system, adducts to the aromatic ring system were twice as abundant as those to the α,β-unsaturated carbonyl. We presume that these differences in adduct formation rates between the open and closed systems can be attributed to the levels of available oxygen in the systems, e.g., higher in the open system and lower in the closed system due to consumption via oxidation. This finding is interesting, but future work is needed to investigate if stochiometric amounts of oxygen are needed or if oxygen is merely a catalyst for these reactions. Additionally, these data suggest that **1a**-, **1b**-, and **1c**-type adducts formed via addition to the aromatic ring after air oxidation to the equivalent o-quinone, may represent the majority of haptenated peptides that trigger immune reactions in vivo*.* However, **2a**-, **2b**-, and **2c**-type adducts still could play an important role and oxygen concentration may influence the adduct type.

Oxidation of catecholic xenobiotics and endogenous metabolites influences their biological effects. Urushiol, for example, is another class of nature-derived catechols (a mixture of compounds which feature a catechol appended with a 15–17 hydrocarbon long chain at the 3 position of the ring). Urushiol is found in poison ivy and poison oak. According to the American Academy of Dermatology, urushiol is responsible for up to 50 million cases of ACD each year in the US. Studies conducted by Castagnoli and co-workers aimed to elucidate the mechanism behind the allergenic potential of urushiol.^29,30^ They demonstrated that urushiol oxidizes quickly to the corresponding o-quinone when in solution at room temperature in the presence of air. The authors conclude that ACD in response to urushiol exposure is likely caused by oxidation (air or enzymatic) to the corresponding quinone derivatives. The resultant quinones are then able to modify endogenous macromolecules. Thus, it is hypothesized that urushiol oxidation is the main mechanism for its skin sensitization potential and ACD.^29,31,32^^,^ Similarly, the oxidation of the catechol in the neurotransmitter dopamine to o-quinone species leads to protein adducts which may contribute to Parkinson’s disease pathology.^33,34^ These aforementioned studies warrant further investigation into the biological effects elicited by catechol-containing compounds. However, the importance of air oxidation of catechol compounds present in propolis has not been investigated in the context of skin sensitization.

Nonenzymatic oxidation of CA and related ferulic acid derivatives to quinone species has been reported.^35,36^ Electrochemical measurements found that pH escalation enhanced the rate of two-electron oxidation of CA to CAQ. It is noted that these authors also found CAQ to be unstable which agrees with our UV–vis experiments that show a rapid decay of CAAEQ and CAPEQ over time. This oxidation process was critical for the generation of peptide adducts in our reaction conditions as no adducts were observed in an oxygen-free environment.

The exact mechanisms for thiol addition to o-quinone systems are still debated in the literature.^29,37^ In addition to nucleophilic attack, radical mechanisms have been put forth to explain the rapid reactivity of thiols and product stereochemistry.^37,38^ It should also be noted that alkyl vs vinyl substitution at the 3 position in the ring influences product stereochemistry. A recent study by Alfieri et al.^37^ investigated the mechanism of the addition of glutathione to dopaquinone, a 3 alkyl-substituted quinone and found that the thiol adds to the 6-position of the ring and not the 2-position, as found in the current study. Interestingly, a previous study exploring the addition of thiols to the quinone derivative formed from CAAE (a 3 vinyl-substituted quinone)^13^ did find that the main thiol adduct is at the 2-position of the ring. This result is in agreement with our current study. The difference between the thiol adducts formed from dopaquinone and the corresponding o-quinones of the caffeates is most likely explained by the differences in the electron distribution caused by the side chains. The dopaquinone has an alkyl group, whereas the caffeates have a conjugated ester/acid. Thus, in the case of the caffeate compounds, addition to the 2-position is likely favored over the 6-position since the 2-position is in further conjugation with the unsaturated ester/acid side chain. Additionally, our results suggest that radical mechanisms induced by air oxidation are important for CA, CAAE, and CAPE reactivity. This is supported by the requirement of oxygen to form peptide adducts as well as CA, CAAE, and CAPE’s reactivity with DPPH. DPPH has been widely used in assays to monitor the presence of labile electrons that can lead to radical formation in different compounds.^21,22^ Additionally, the formation of the adducts **2a**–**c** only in the presence of oxygen and not under inert conditions implies that they are formed via a thiol–ene mechanism rather than a Michael addition (Figure 4), highlighting the importance of air oxidation for the sensitizing potential of the caffeic acid derivatives.^39^ Given these results, more detailed mechanistic studies could be conducted to verify the presence of transitory radical species in thiol reactions with CA, CAAE, and CAPE. However, such work is beyond the scope of this study.

Simplified in vitro and *in chemico* assays, such as the ones employed in this study, are excellent strategies to gain mechanistic insights into complex processes such as hapten formation. However, such approaches may not fully recapitulate the complexity of an in vivo system. For example, metabolic processes can detoxify or activate xenobiotics and are likely important in propolis skin sensitization. Specifically, glutathione present in high concentrations in living cells can protect proteins by scavenging reactive electrophiles and radical species. Future work will utilize physiologically relevant skin model systems to verify the presence of the hapten peptides uncovered here. Additionally, the structural information obtained from our work can be used to study whether different adducts (**1a–c** vs **2a–c**) elicit different immune responses. Such information may be useful in understanding the development of contact allergy to haptens in natural products and in developing tools for diagnosis of contact allergy to propolis, a natural product and complex mixture.

## 5 Conclusions

CA, CAAE, and CAPE are major components of poplar-type propolis and are known to cause allergic skin reactions. Classification of these compounds into different sensitization categories utilizing the kDPRA was not successful, highlighting the limitations of the assay. Employment of LC-ESI-HRMS for investigation of adduct formation of these compounds with the synthetic peptide Ac-PHCKRM provided structural insights into CA, CAAE, and CAPE peptide adducts. The importance of air oxidation for activation of these compounds to the ultimate reactive species was revealed based on the detected peptide adducts. This signifies that these compounds are in fact prehaptens. Collectively, our studies contribute to the understanding of the structures and formation mechanisms of adducts formed from CA derivatives, which is critical in understanding the topology and generation of hapten-modified peptides/proteins that could drive skin sensitization toward propolis.

## Supporting Information Available

kDPRA OECD procedure and obtained plots, depletion values for all time points and concentrations tested during both versions of the kDPRA, exact masses and *m*/*z* monitored in the LC-HRMS analysis, LC–ESI^+^–HRMS structural analysis of CAAE and CAPE adducts, and NMR spectra of oxidized CA with NAC (PDF)
