# Supplementary material for: Investigation into Propolis Components Responsible for Inducing Skin Allergy: Air Oxidation of Caffeic Acid and Its Esters Contribute to Hapten Formation
Source: Chem Res Toxicol. 2023 May 15;36(6):859–69. doi: 10.1021/acs.chemrestox.2c00386 (PMC10283018; doi:10.1021/acs.chemrestox.2c00386)
Supplement: Supplementary file 1 — tx2c00386_si_001.pdf [file tx2c00386_si_001.pdf]

Investigation into Propolis Components Responsible for Inducing Skin Allergy: Air Oxidation of Caffeic Acid and its Esters Contribute to Hapten Formation

*Lorena Ndreu[1], Alexander K. Hurben,[2] Gunnar S A Nyman[3,4], Natalia Y. Tretyakova,[2] Isabella Karlsson[1], Lina Hagvall[4,5]**

[1]Department of Environmental Science, Stockholm University, Stockholm, Sweden.

[2]Department of Medicinal Chemistry and the Masonic Cancer Center, University of Minnesota, Minneapolis, Minnesota 55455, United States.

[3]Department of Dermatology and Venereology, Region Västra Götaland, Sahlgrenska University Hospital, Gothenburg, Sweden.

[4]Department of Dermatology and Venereology, Institute of Clinical Sciences, Sahlgrenska Academy, University of Gothenburg, Gothenburg, Sweden.

[5]Department of Occupational and Environmental Medicine, Lund University, Lund, Sweden.

Contents

1.1 OECD Kinetic Direct Peptide Reactivity Assay (kDPRA) 3

kDPRA Acceptance Criteria...4

Figure S1: kDPRA results for A) CA, B) CAAE, and C) CAPE. The natural log of unreacted Ac-RFAACAA peptide (100 – relative peptide depletion (DP) %) values are plotted against the increasing concentrations of CA, CAAE, and CAPE at specified time points...............................5

Table S1 6

Table S2 7

Table S3: Exact mass (Da) and m/z of the singly and doubly charged precursor ions of the CA, CAAE, CAPE expected peptide adducts. 8

Figure S2: HPLC-ESI+-HRMS structural analysis of CAAE and Ac-PHCKRM peptide adducts after incubation of the peptide with 5 times molar excess of CAAE, in 1:1 PB (pH 7.4) and ACN for 5 h prior to LC-HRMS analysis. A & B) MS2 spectrum of 1a and 1b with annotated b and y ions. C & D). Structures of the y ions carrying the CA adduct. 9

Figure S3: HPLC-ESI+-HRMS structural analysis of CAPE and Ac-PHCKRM peptide adducts after incubation of the peptide with 5 times molar excess of CAPE, in 1:1 PB (pH 7.4) and ACN for 5 h prior to LC-HRMS analysis. A & B) MS2 spectrum of 1a and 1b with annotated b and y ions. C & D). Structures of the y ions carrying the CA adduct. 10

Figure S4. NMR spectra of 10 mM CA, 10 mM CA and 2.5 eq of NaIO4 (10 min reaction), or 10 mM CA reacted with 1 eq of NaIO4 and 5 eq of NAC, shown in from top to bottom, respectively. Reactions were performed in a mixture of MeOD and deuterated PBS (pH 7.4). Labels above proton resonances denote corresponding molecular labels. 11

Figure S5. NMR spectra of reaction time course between 10 mM CA (A), CAAE (B), or CAPE (C) and 10 mM NAC in a 1:1 mixture of DMSOd6 and deuterated PBS (pH 7.4). Spectra were acquired 18 hours after reaction initiated. NAC adduct proton resonances are denoted by * labels. D) Pictures of NMR tubes following the 18 h reaction. The insoluble precipitate formed in the CAPE reaction may explain the lack of observable adduct peaks...13

1.1 OECD Kinetic Direct Peptide Reactivity Assay (kDPRA)

Assay Protocol

CA, CAAE, and CAPE were dissolved in ACN to yield stock solutions at a concentration of 20 mM. These stock solutions were used to prepare a dilution series of 20, 10, 5, 2.5 and 1.25 mM for each compound. 120 µL of a 0.667 mM peptide (Ac-RFAACAA) stock solution in pH 7.5 phosphate buffer were added to a black 96 well plate. Next, compounds were added to the wells to provide a final 0.5 mM peptide concentration and 5, 2.5, 1.25, 0.625 and 0.3125 mM respectively of the tested compounds. The final volume in each well was 160 µL. The plates were sealed with a gas-tight adhesive foil and shaken for 5 min and the reactions were allowed to proceed for 10, 30, 90, 150, 210, or 1440 min at 25 ± 2.5 ˚C. The reactions were stopped by addition of 40 µL of a 3 mM solution of MBrB, which reacts rapidly with unmodified cysteine moieties of the peptide Ac-RFAACAA to generate a fluorescent complex. The plate was shaken for 5 min prior to measuring fluorescence with a TECAN® Infinite M Nano Photometer and Plate Reader, equipped with a Magellan™ Tracker Software (Ex: 390 nm, Em 480 nm). Cinnamic aldehyde, a substance known to cause peptide depletion with a constant kinetic rate was used as a

positive control (PC). Other controls included peptide only as a negative control (NC) and respective test-substance only as a substance control (SC).

Data Evaluation

The relative peptide depletion (DP) % was calculated for each compound tested through **Equation 1**.

$$(1)\ DP = (1 - \frac{Corrected\ Sample\ Value}{Corrected\ Mean\ of\ Negative\ Control}) \times 100\%$$

The corrected sample value is taken as the measured sample fluorescence value minus the value of the test SC. The corrected mean value of the NC is the average of 12 NC measurements minus the mean of 12 background measurements controls (BC). The obtained DP % values are then converted to 1n(100-DP) for kinetic analysis. The slope and correlation values are generated from plots of 1n(100-DP) versus the different concentrations for 10, 30, 90, 150, 210, and 1440 min reaction time points.

The observed reaction kinetic constant k_{obs} (mM^{-1}) corresponds to the slope for each respective reaction time point.

The correlation over all concentrations, is calculated from the k_{obs} value of each time point and expressed as the reaction kinetic constant k per M and sec (M^{-1} s^{-1}) as shown in **Equation 2**. [14]

$$(2)\ k = k^{obs} \times \frac{1000}{(60 \times time\ point\ [\text{min}])}$$

For each time point with a correlation >0.9, the common logarithm of k (log k) is calculated. The log k_{max} (highest k value) and the respective time point are reported.

kDPRA Acceptance Criteria

The results obtained from the analysis of a test 96-well plate are considered valid, if the following criteria are met: (1) The log k value of the positive control at 90 min falls between the range: -1.75 M-1s-1 to -1.40 M-1s-1. Note, if the value at 90 min cannot be obtained, the value at 150 min is used instead. In this case the value at 150 min should be between the range: -1.90 M-1s-1 to -1.45 M-1s-1. (2) The coefficient of variation of in the 12 NC measurements in a given plate is < 12.5% for at least 5 of the 6 time points.

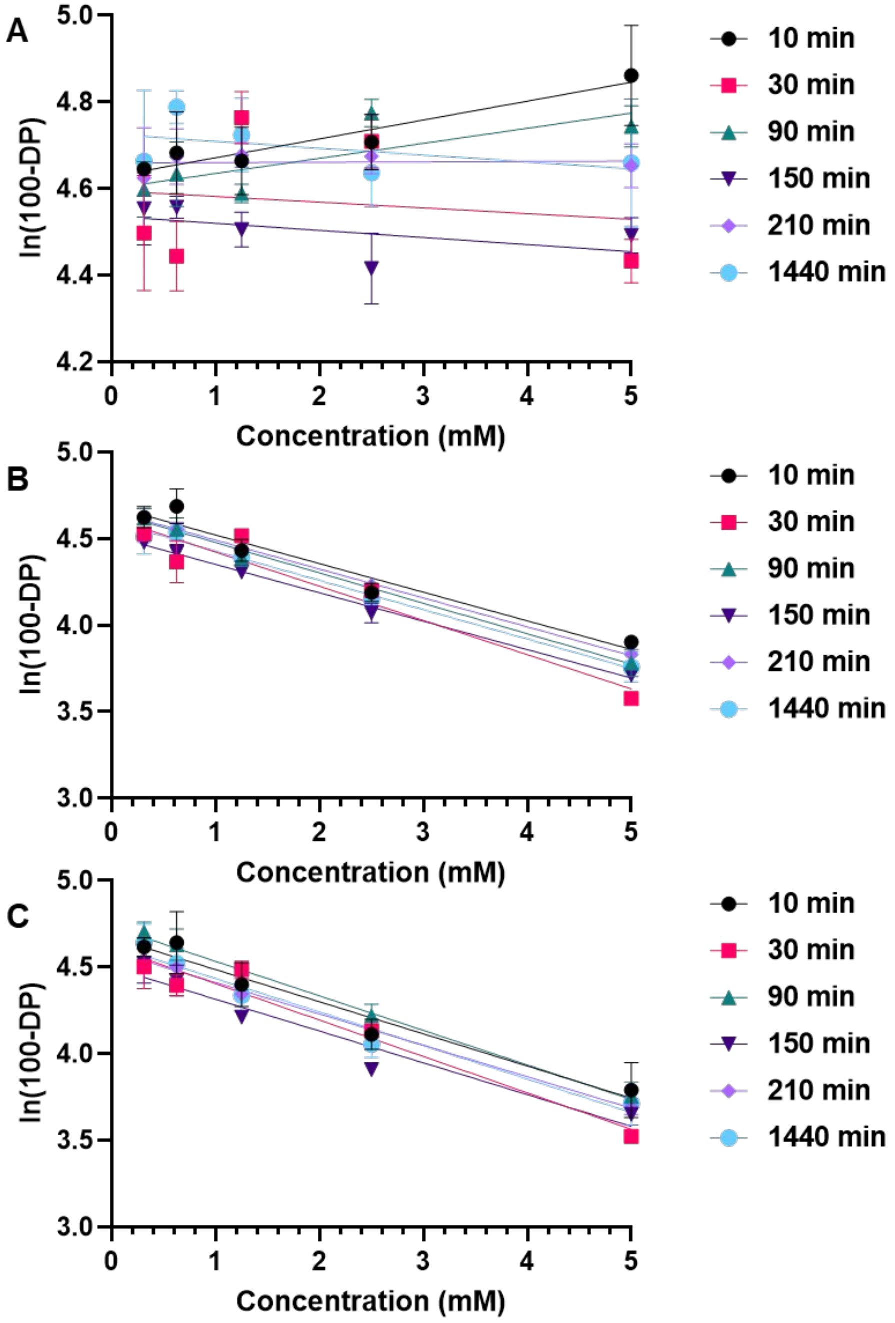

Figure S1: **k**DPRA results for A) CA, B) CAAE, and C) CAPE. The natural log of unreacted Ac-RFAACAA peptide (100 – relative peptide depletion (DP) %) values are plotted against the increasing concentrations of CA, CAAE, and CAPE at specified time points.

Table S1: Depletion values for each time point and concentration tested during the kDPRA performed following the OECD guideline.

kDPRA						
		**Caffeic acid**				
		Depletion at concentration [final concentration mM]				
		5	2.5	1.25	0.625	0.3125
t [min]	10	1.00	1.00	1.00	1.00	1.00
	30	15.74	1.00	1.00	14.69	9.67
	90	1.00	1.00	1.61	1.00	1.00
	150	10.64	17.06	9.44	4.67	4.78
	210	1.00	1.00	1.00	1.00	1.00
	1440	1.00	1.00	1.00	1.00	1.00
		**Caffeic acid 1,1-dimethyl allyl ester**				
		dp at concentration [final concentration mM]				
		5	2.5	1.25	0.625	0.3125
t [min]	10	50.38	33.88	15.67	1.00	1.00
	30	64.19	33.11	8.23	20.78	7.33
	90	56.01	31.85	19.23	4.50	1.00
	150	59.04	41.24	25.61	16.64	11.72
	210	53.84	31.08	15.07	4.72	1.00
	1440	56.98	36.40	19.19	6.19	8.45
		**Caffeic acid phenethyl ester**				
		dp at concentration [final concentration mM]				
		5	2.5	1.25	0.625	0.3125
t [min]	10	55.30	38.75	18.01	1.00	1.00
	30	66.07	37.64	11.08	18.80	9.25
	90	57.30	31.86	16.00	1.00	1.00
	150	61.46	50.14	32.48	16.35	7.70
	210	59.66	38.07	22.83	10.12	5.77
	1440	58.82	42.20	23.22	7.66	1.00

Table S2: Depletion values for each time point and concentration tested during the modified kDPRA performed.

Modified kDPRA						
		Caffeic acid				
		Depletion at concentration [final concentration mM]				
		5	2.5	1.25	0.625	0.3125
t [min]	10	15.41	4.69	1.00	1.00	1.00
	30	15.64	4.56	5.00	1.33	1.00
	90	12.02	1.00	1.00	1.00	1.00
	150	31.59	16.93	10.81	4.40	4.79
	210	26.37	11.62	1.20	1.00	1.00
	300	18.34	1.00	1.00	1.00	1.00
		Caffeic acid 1,1-dimethyl allyl ester				
		dp at concentration [final concentration mM]				
		5	2.5	1.25	0.625	0.3125
t [min]	10	47.45	30.72	17.78	10.09	7.66
	30	45.49	29.71	17.75	9.78	3.67
	90	43.71	24.33	14.05	6.19	1.98
	150	51.35	32.92	21.69	14.66	14.32
	210	47.53	29.33	17.52	6.95	3.43
	300	47.30	23.64	12.62	1.00	1.00
		Caffeic acid phenethyl ester				
		dp at concentration [final concentration mM]				
		5	2.5	1.25	0.625	0.3125
t [min]	10	54.18	38.58	25.82	20.56	17.83
	30	54.00	39.17	27.45	19.21	12.72
	90	58.88	43.42	32.40	23.46	19.58
	150	54.85	37.85	25.85	17.83	18.08
	210	56.81	40.90	30.69	21.08	11.04
	300	61.52	45.01	37.05	27.49	16.97

Table S3: Exact mass (Da) and m/z of the singly and doubly charged precursor ions of the CA, CAAE, CAPE expected peptide adducts.

Adduct	Exact Mass (Da)	Singly Charged Ion (m/z)	Doubly Charged Ion (m/z)
1a	990.4052	991.4130	496.2104
2a	992.4208	993.4286	497.2182
1b	1058.4678	1059.4756	530.2417
2b	1060.4834	1061.4912	531.2495
1c	1094.4678	1095.4756	548.2417
2c	1096.4834	1097.4912	549.2495

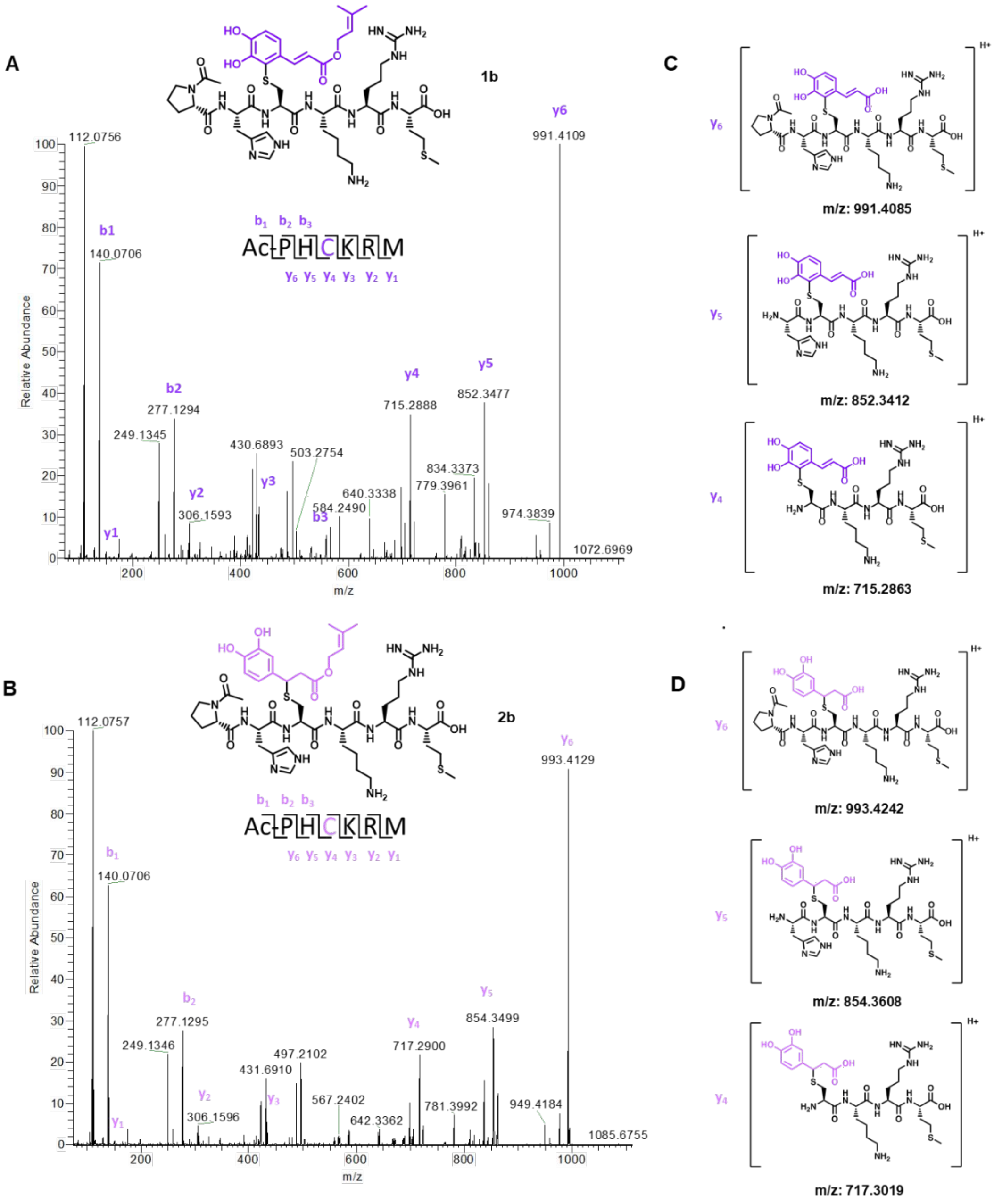

Figure S2: HPLC-ESI+-HRMS structural analysis of CAAE and Ac-PHCKRM peptide adducts after incubation of the peptide with 5 times molar excess of CAAE, in 1:1 PB (pH 7.4) and ACN for 5 h prior to LC-HRMS analysis. A & B) MS2 spectrum of 1a and 1b with annotated b and y ions. C & D). Structures of the y ions carrying the CA adduct.

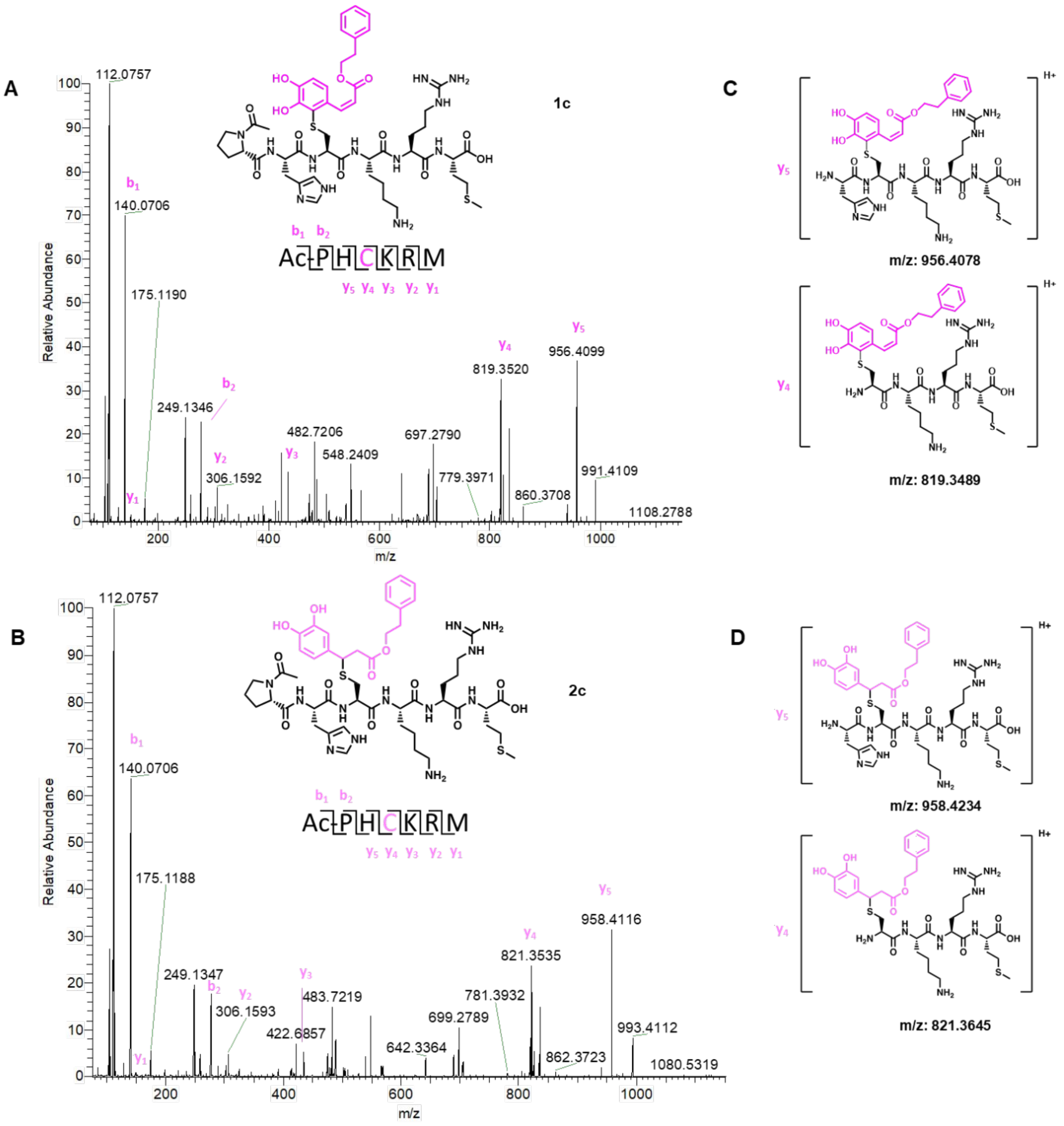

Figure S3: HPLC-ESI+-HRMS structural analysis of CAPE and Ac-PHCKRM peptide adducts after incubation of the peptide with 5 times molar excess of CAPE, in 1:1 PB (pH 7.4) and ACN for 5 h prior to LC-HRMS analysis. A & B) MS2 spectrum of 1a and 1b with annotated b and y ions. C & D). Structures of the y ions carrying the CA adduct.

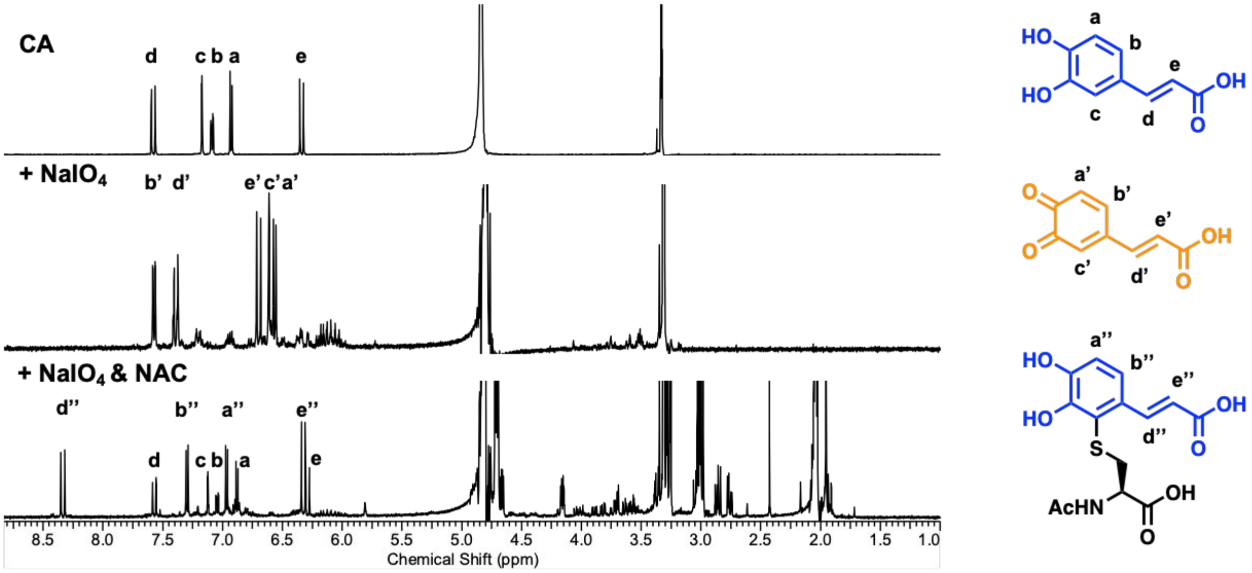

Figure S4. NMR spectra of 10 mM CA, 10 mM CA and 2.5 eq of NaIO4 (10 min reaction), or 10 mM CA reacted with 1 eq of NaIO4 and 5 eq of NAC, shown in from top to bottom, respectively. Reactions were performed in a mixture of MeOD and deuterated PBS (pH 7.4). Labels above proton resonances denote corresponding molecular labels.

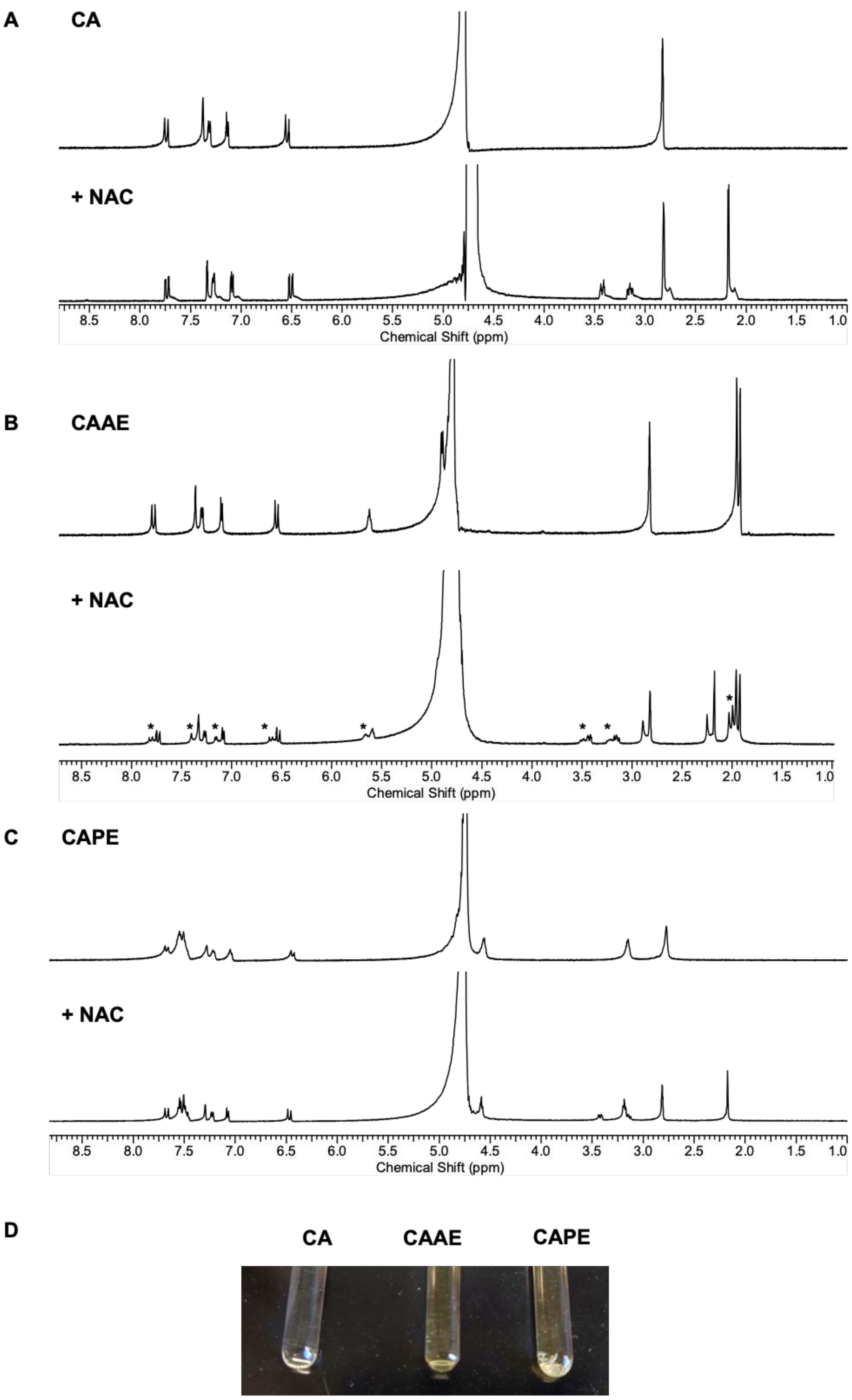
A
CA
+ NAC
8.5 8.0 7.5 7.0 6.5 6.0 5.5 5.0 4.5 4.0 3.5 3.0 2.5 2.0 1.5 1.0
Chemical Shift (ppm)
B
CAAE
+ NAC
8.5 8.0 7.5 7.0 6.5 6.0 5.5 5.0 4.5 4.0 3.5 3.0 2.5 2.0 1.5 1.0
Chemical Shift (ppm)
C
CAPE
+ NAC
8.5 8.0 7.5 7.0 6.5 6.0 5.5 5.0 4.5 4.0 3.5 3.0 2.5 2.0 1.5 1.0
Chemical Shift (ppm)
D
CA
CAAE
CAPE

Figure S5. NMR spectra of reaction time course between 10 mM CA (A), CAAE (B), or CAPE (C) and 10 mM NAC in a 1:1 mixture of DMSOd6 and deuterated PBS (pH 7.4). Spectra were acquired 18 hours after reaction initiated. NAC adduct proton resonances are denoted by * labels. D) Pictures of NMR tubes following the 18 h reaction. The insoluble precipitate formed in the CAPE reaction may explain the lack of observable adduct peaks.
